# MEASURING PROFESSIONAL KNOWLEDGE OF LATER LIFE DEPRESSION

**DOI:** 10.1093/geroni/igac059.1087

**Published:** 2022-12-20

**Authors:** Abby Laine, Ann Steffen

**Affiliations:** University of Missouri-St. Louis, St. Louis, Missouri, United States; University of Missouri-St. Louis, St. Louis, Missouri, United States

## Abstract

Research linking behavioral health providers’ knowledge of later life depression to their clinical practices and patient outcomes is restricted by outdated measures. A multi-stage study was conducted to ameliorate this gap. Qualitative interviews with geropsychology content experts (N = 5) generated 49 true/false items capturing relevant concepts in later life depression, including brain health concerns. Additional content experts (N = 10) completed the questionnaire and a card sort task placing items into categories (Psychopathology, Assessment/Diagnosis, Treatment, Other) to understand measurement structure. This resulted in retention of 42 items. A random pool of licensed social workers (N=900) were mailed the survey packet with option to complete via return mail or online. This presentation will review scale psychometrics, reliability, and construct validity among MSWs from diverse clinical backgrounds. Associations between participant characteristics and knowledge of cognitive and other depressive symptoms will also be highlighted.

